# Inhibition of sialidase activity and cellular invasion by the bacterial vaginosis pathogen *Gardnerella vaginalis*

**DOI:** 10.1007/s00203-018-1520-4

**Published:** 2018-05-18

**Authors:** G. Govinden, J. L. Parker, K. L. Naylor, A. M. Frey, D. O. C. Anumba, G. P. Stafford

**Affiliations:** 1 Integrated BioSciences Group, School of Clinical Dentistry, University of Sheffield, Sheffield, S10 2TA UK; 2 Academic Unit of Reproductive and Developmental Medicine, Department of Oncology and Metabolism, University of Sheffield, Sheffield, S10 2TA UK; 3 Department of Obstetrics and Gynaecology, Jessop Wing, Sheffield Teaching Hospitals, Tree Root Walk, Sheffield, S10 2ST UK

**Keywords:** Sialidase, Bacterial vaginosis, Microbiology, Epithelial invasion

## Abstract

Bacterial vaginosis is a genital tract infection, thought to be caused by transformation of a lactobacillus-rich flora to a dysbiotic microbiota enriched in mixed anaerobes. The most prominent of these is *Gardnerella vaginalis* (GV), an anaerobic pathogen that produces sialidase enzyme to cleave terminal sialic acid residues from human glycans. Notably, high sialidase activity is associated with preterm birth and low birthweight. We explored the potential of the sialidase inhibitor Zanamavir against GV whole cell sialidase activity using methyl–umbelliferyl neuraminic acid (MU-NANA) cleavage assays, with Zanamavir causing a 30% reduction in whole cell GV sialidase activity (*p* < 0.05). Furthermore, cellular invasion assays using HeLa cervical epithelial cells, infected with GV, demonstrated that Zanamivir elicited a 50% reduction in cell association and invasion (*p* < 0.05). Our data thus highlight that pharmacological sialidase inhibitors are able to modify BV-associated sialidase activity and influence host–pathogen interactions and may represent novel therapeutic adjuncts.

## Introduction

Bacterial vaginosis (BV) is a prevalent condition characterised by vaginal irritation and malodour. It is hypothesised in BV that the vaginal microbiome alters from a rich *lactobacilli flora*; to a dysbiotic population rich in mixed anaerobes with *Gardnerella vaginalis* (GV) prominent (Ling et al. 2010; Brocklehurst et al. 2013). Often asymptomatic, BV is a risk factor for Pelvic Inflammatory disease, sexually transmitted infections and post-operative infections (Cauci et al. 2005; Allsworth and Peipert 2007; Lewis et al. 2013; Schwebke et al. 2014). BV increases risk of preterm birth (PTB), with reduction in lactobacilli and increased mixed anaerobes strongly correlating with increased PTB risk (Cauci et al. 2005; Nelson et al. 2009; Marrs et al. 2012; Marconi et al. 2013; Bretelle et al. 2015). Furthermore, blanket antibiotic treatment does not reduce the incidence of PTB and so a more targeted approach may be required (McDonald et al. 2007; Marrs et al. 2012; Romero et al. 2014).

One prominent trait of the dysbiotic microbiota in BV is production of high levels of sialidase enzymes produced by bacteria that act to release sialic acid, the terminal glycan on many glycoproteins in secretions and on mucosal cell surfaces- including vaginal mucous (Severi et al. 2007; Stafford et al. 2011; Lewis et al. 2012; Hardy et al. 2017). This sialic acid is used by pathogens as a mechanism of adherence to cellular and inert surfaces, as a source of nutrition and also modifies the normal mucus barrier and immune response (Amith et al. 2009, 2010; Stafford et al. 2011; Lewis et al. 2012, 2013; Vick et al. 2014). Evidence suggests BV in conjunction with high sialidase activity is predictive of PTB and low birthweight, while BV alone is not (Cauci et al. 2005).

The most prominent and well-studied bacterium in BV is *G. vaginalis*; a Gram Variable anaerobic coccobacillus with a thin Gram-positive type cell wall (Cauci et al. 2005; Marrs et al. 2012; Lewis et al. 2013; Castro et al. 2015). Many strains of *G. vaginalis* are sialidase positive, with activity likely related to pathogenesis and virulence and potentially for survival in this environment (Cauci et al. 2005; Lopes Dos Santos Santiago et al. 2011; Gilbert et al. 2013; Lewis et al. 2013; Hardy et al. 2017). In addition, genome sequencing of *G. vaginalis* strains has revealed three main clade ecotypes, with all except clade 3B containing putative sialidase genes (Cornejo et al. 2018). Given the prominence and likely importance of sialidase activity in BV infections, we set out in this pilot study to test whether the sialidase inhibitor Zanamivir (Relenza^®^), might inhibit GV sialidase activity, and determine how this might affect its reported interaction with human cervical epithelial cells in vitro (Hardy et al. 2017).

## Methods

### Bacterial strains and growth media

*Gardnerella vaginalis*, strains JCP8017A and JCP8066 [clades 2A and 2B, respectively (Cornejo et al. 2018)], obtained through BEI Resources, NIAID, NIH as part of the Human Microbiome Project, and checked on arrival microscopically and by 16s rRNA sequencing. These were cultured on Casman Agar^®^ (BD, UK) with 10% oxalated horse blood (Oxoid, Fisher Scientific, UK) in a Don Whitley MiniMacs anaerobic cabinet (10% CO_2_, 10% H_2,_ and 80% N_2_) at 37 °C for 72 h.

### Whole cell sialidase assays

3-day-old bacterial colonies were transferred into 1 ml of sterile phosphate-buffered saline (PBS, pH 7.4) and centrifuged at 10,000×*g* for 1 min and washed three times before dilution to an *A*_600_ of 1.0 with 10 µl added to each reaction mix containing 0.2 mM MUNANAC (Carbosynth), in 20 mM Tris–HCl (pH 7.4) or 100 mM sodium acetate (pH 5.5): total volume 100 µl, for 1 h. Reactions were incubated at 37 °C in an anaerobic cabinet in the presence or the absence of Zanamivir (Relenza^®^) 10 mM (a concentration previously established to inhibit oral anaerobe sialidases, Stafford, unpublished). Readings of fluorescence (excitation 370 nm emission 420 nm) were measured as indicated (REF).

### Host–pathogen invasion assay

HeLa cells were cultured in a sterile cabinet using Dulbecco’s Modified Eagle’s Media (DMEM) (Sigma, Poole) with 10% fetal calf serum (Sigma, Poole), 1% 2 mM glycerine (Bio Whitlaker DE14-870FH), and 1% Pen/strep (10,000 units Penicillin, 10 mg Streptomycin per ml) (Sigma, Poole). Cells were grown to 70–80% confluence and split every 3–4 days using trypsin EDTA (Sigma, Poole).

Invasion assays were performed as previously described (Naylor et al. 2017). In brief, 2.0 × 10^5^ HeLa cells were seeded in 24-well tissue-culture plates overnight in media without pen/strep, before washing with PBS and incubated for 1 h at 37 °C, 5% CO_2_ in DMEM with 2% bovine serum albumin (BSA) (Sigma, Poole). Following incubation, BSA solution was removed and cells washed in PBS, before GV JCP8066 was added at a multiplicity of infection (MOI) of 1:100 with or without Zanamivir (10 mM) for 90 min at 37 °C 5% CO_2_. Cells were then washed with PBS to remove unattached bacteria. For total bacterial counts, cells were lysed before being plated on Casman agar using the Miles–Misra method (Miles et al. 1938). To determine bacterial invasion counts, wells were treated with metronidazole 200 µg/ml to kill extracellular bacteria and incubated for 1 h at 37 °C, and 5% CO_2_ conditions we have tested and shown to reduce viable counts of GCV8066 by 3-logs (not shown). Following incubation, cells were lysed and colonies counted, before invasion etc. calculated as a percentage of the viable count of the initial innoculum.

All experiments were repeated in technical triplicate with three biological repeats. Statistical difference between groups was established using Students *t* test. Cytotox 96 assays^®^ (Promega, UK) were performed to confirm non-toxicity of the drugs (data not shown).

## Results

### Inhibition of whole *G. vaginalis* cell sialidase activity by Zanamivir

Sialidase assays examined if bacterial sialidase activity could be directly inhibited by the sialidase inhibitor Zanamivir, marketed as Relenza^®^. This was chosen, because we aimed to study a drug used clinically with a well-established safety profile. First, Zanamavir is approved for clinical use by the FDA for influenza treatment. Second, unlike oseltamivir phosphate (active agent in TamiFlu^®^), application as a topical agent may be feasible as TamiFlu^®^ requires liver metabolism into an active form, while Zanamivir is effective when used in a topical form and supplied as an aerolised powder for inhalation into the lungs of influenza sufferer. Moreover, it is safe in pregnancy and has been used topically safely in man (Hayden et al. 1997; Xie et al. 2013; Jefferson et al. 2014).

**Fig. 1:**
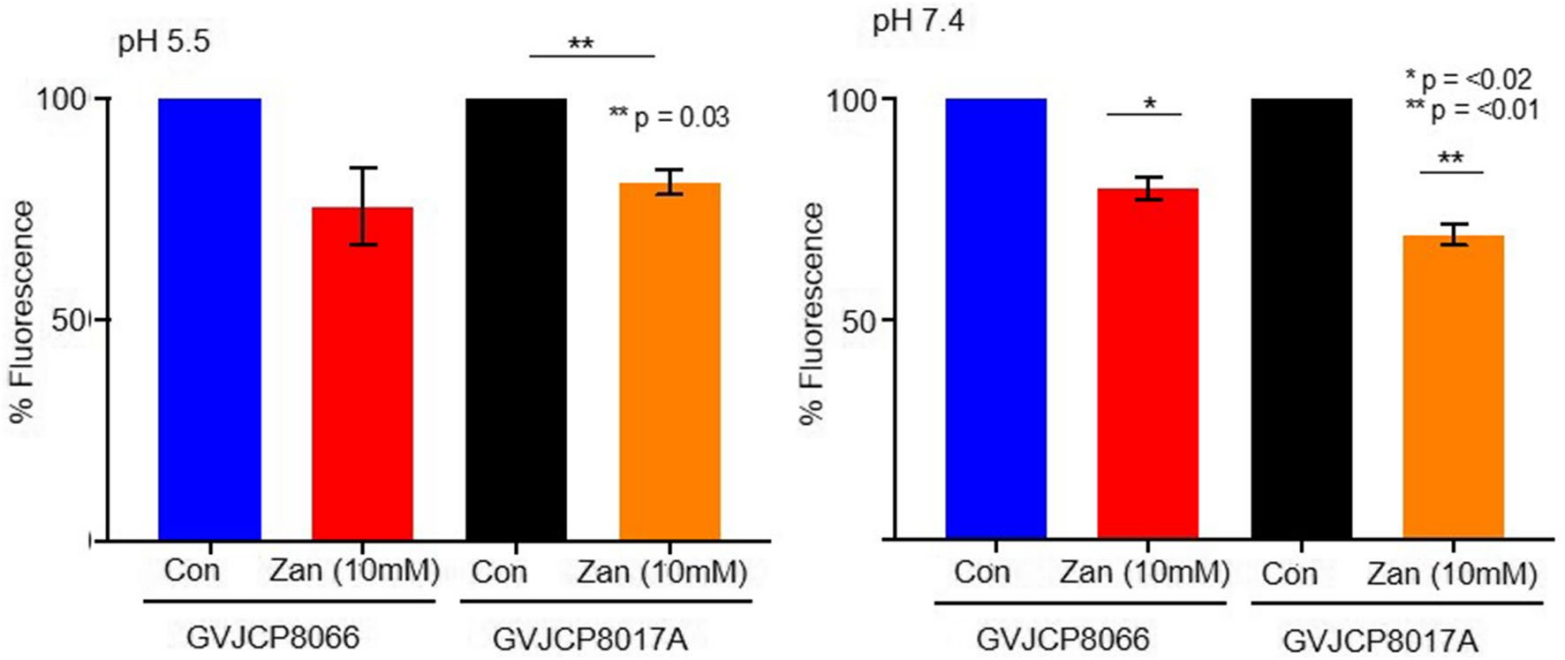
Inhibition of whole cell *G. vaginalis sialidase* by Zanamivir (10 mM). Whole bacteria (strains as indicated) were incubated with Zanamivir in the presence of MU-NANA (0.2 mM) as described for 1 h. Sialidase activity was measured using readings of fluorescence (excitation 370 nm emission 420 nm). Activity was calculated as a percentage of sialidase activity seen with no inhibitor (Con). Assays were conducted at a pH 7.4 and pH 5.5 as indicated. Students’ *t* test was used to calculate significant differences between con and drug, and noted in A (*n* = 3). Error bars are ± standard error of the mean (SEM). Fig produced using GraphPad Prism

We tested inhibition of two sialidase positive strains of *G. vaginalis*, namely, GV JCP8066 and GV 8017A, which are members of GV clade 2A and 2B (Lewis et al. 2013; Cornejo et al. 2018) in whole cell bacterial sialidase assays (where both cell associated and enzyme released by cells during the assay time course would be present) using 10 mM Zanamivir at both neutral and acidic pH—pH 5.5 was chosen as it had previously been shown to be the optimum pH for this enzyme (von Nicolai et al. 1984); notable vaginal pH is normally around 4.5 and is raised in BV (Cauci et al. 2005; Gilbert et al. 2013). In both *G. vaginalis* strains, and at both pH values tested, Zanamivir significantly (*p* < 0.05), reduced sialidase activity of whole bacterial *G. vaginalis* cells up to 30% at 10 mM (N.B. this was the highest concentration achievable in solution in these assay conditions, and a dose achievable potentially clinically, where a 10 mg dose is given 4× per day) (Fig. 1).

### Effect of Zanamivir on bacterial invasion of human cervical cells

**Fig. 2:**
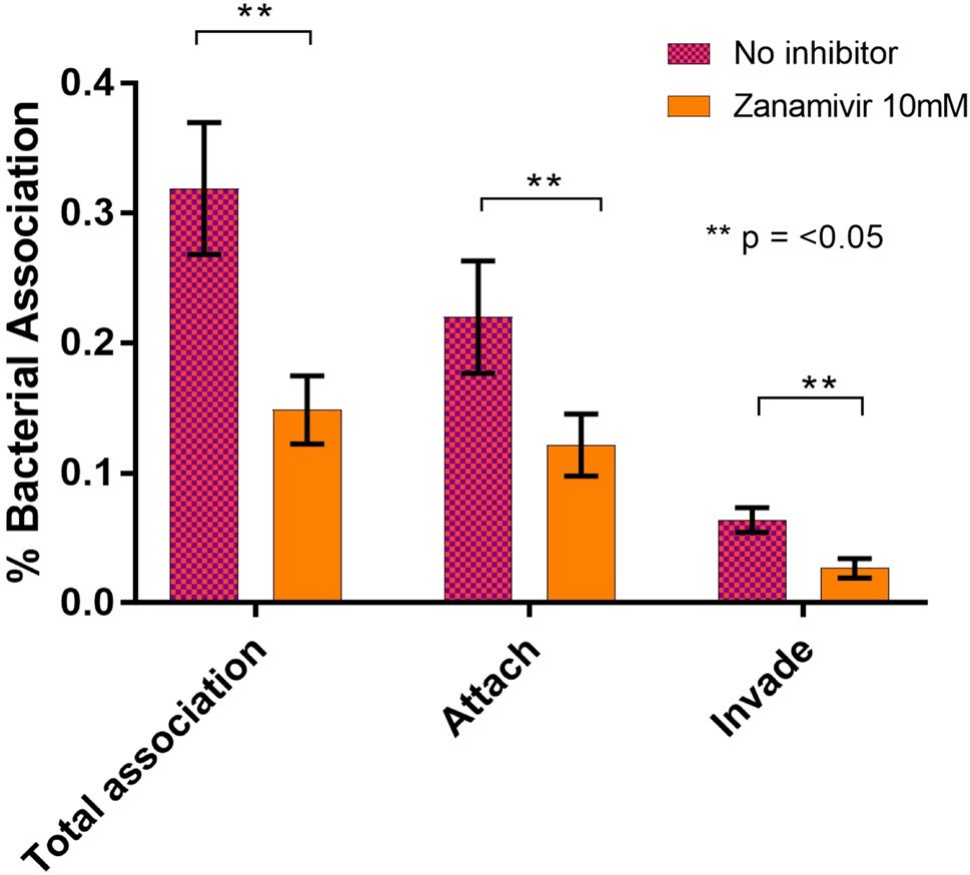
Influence of Zanamavir on host–pathogen interaction of *G. vaginalis* with cervical cells. HeLa cell monolayers were infected with *G. vaginalis* strain JCP8066. Total association, cell membrane adhered (attached), and invaded cells were calculated as described in methods. Bacteria were counted as colony forming units (CFU) and calculated as a percentage of viability from the bacterial inoculum CFUs (*n* = 3). Students’ *t* test used to calculate significance and noted as **p < 0.05. Error bars are ± SEM. Fig produced using GraphPad Prism

We then utilised strain GV JCP8066 in antibiotic protection assays chosen, since in preliminary studies, it displayed the most stable growth and aerotolerance in preliminary studies (data not shown). We performed invasion assays at an MOI of 1:100, and observed association and invasion rates of approximately 1%; typical of invasion rates our laboratory commonly observes for oral anaerobes such as *Porphyromonas gingivalis, Tannerella forsythia*, and *Fusobacterium* spp. (Naylor et al. 2017). In contrast, assays performed in the presence of Zanamivir, displayed a dramatic, and statistically significant reduction in levels of total association (2.2-fold, *p* = 0.03), adhesion (1.95-fold, *p* = 0.04), and invasion (2.4-fold, *p* = 0.03) (Fig. 2). Indicating Zanamivir can reduce cellular internalisation of *G. vaginalis* by approximately twofold under the conditions tested (Fig. 2).

## Discussion

In this study, we established that Zanamivir reduces two facets of the virulence of the BV-associated pathogen *G. vaginalis*, namely, its sialidase activity and ability to invade Human cells. However, we also acknowledge here that our sialidase activity assays only focussed on an artificial substrate, albeit a widely used compound to assess sialidase activity, and that in vivo sialidase act upon a wide range of more complex glycans with varying levels of preference and activity. In addition, it may be that the inhibitor used here also had varied inhibitory activity on these varying substrate–enzyme interactions, a picture that would be improved by performing further assays with complex glycans and a range of strains.

This observation is similar to other sialidase positive pathogens, where sialidase activity is linked to the ability to adhere to glycoproteins and invade (and thus potentially damage) epithelial cells (Honma et al. 2011; Roy et al. 2011). Indeed, *G. vaginalis* and an increased sialidase activity from vaginal samples are associated with disease and increased risk of PTB (Simhan et al. 2003; Cauci et al. 2005; Bretelle et al. 2015). In addition, symptomatic BV cases tend to be associated sialidase positive readings of swabs (Lopes Dos Santos Santiago et al. 2011). Evidence thus suggests that sialidase activity of these strains has evolved as an adaptation to life in a glycan rich niche and may aid pathogenesis. Thus directly targeting pathogens that possess sialidase activity might be an alternative approach to antibiotic treatment for BV during pregnancy, since antibiotics have been shown to have no effect on the incidence of PTB (McDonald et al. 2007).

Our data indicate that Zanamavir reduces sialidase activity, but also impairs the ability of *G. vaginalis* to invade human cells. This may have import in BV, since the ability to enter epithelial cells is considered both a strategy to evade the immune system, but may also lead to formation of transient intracellular reservoirs of bacteria that can recolonize the mucosa and microbiota (Marrs et al. 2012). Of note is also that any intracellular bacteria are also able to evade antibiotic treatment requiring new approaches to treat them or preventing their invasion (Ranjan et al. 2012).

Another potential beneficial factor to sialidase inhibition is that it is now clear that sialidases can influence innate immune responses to bacteria by modulating the levels of sialic acid on key innate immune receptors such as Toll-like receptors 2 and 4 (Amith et al. 2010). This is the case for both bacterial (Amith et al. 2009) and human sialidases (NEU1 and NEU3), both of which are inhibited by Zanamavir, but which also contain more complex glycans (Hata et al. 2008). Thus, by inhibiting either bacterial or human sialidases pharmacologically, there is the potential to reduce inflammation. In addition, sialidase targets IgA-key to inhibiting adhesion of bacteria at mucosal surfaces, meaning that inhibition of sialidase in vivo may also decrease bacterial adhesion by maintaining functional IgA (Lewis et al. 2012).

The final effect of reducing sialidase levels would be to reduce the amount of free sialic acid for bacterial growth, and hence starving certain bacteria of a key nutrient. This potential to decrease free sialic acid may have other knock-on effects with evidence from mouse models that sialidases may have a role in promoting growth of *group B streptococci* by releasing free sialic acid, and potentially the propagation of ascending genital tract infection (Pezzicoli et al. 2012; Gilbert et al. 2013). Given the poly-microbial nature of BV, it seems plausible that targeting this metabolic activity has the potential to manipulate the microbiota to suppress sialidase positive BV pathogens and potentially promote rebiosis to a lactobacillus dominate community, although further experiments in a poly-microbial model of the vaginal microbiota are necessary.

However, we are aware that our study is limited, with a need for expansion of the repertoire of species used, range of drugs tested and potentially using primary vaginal cells. In addition, as mentioned above, sialidase activity can influence innate immune responses and it would be of interest to establish whether inhibitors reduce pro-inflammatory cytokine production, as is the case for *S. pneumoniae* (Paulson and Kawasaki 2011).

In conclusion, we have established the anti-influenza drug Zanamivir can inhibit GV sialidase and influence host–pathogen interactions in vitro, suggesting future directions for investigation and potential of these drugs as topically applied adjuncts for BV treatment and reduction of associated morbidities.
